# Pathology of Rare Umbilical Lesions: A Case Series and Literature Review

**DOI:** 10.7759/cureus.107191

**Published:** 2026-04-16

**Authors:** Iqbal Singh, Rugvi Patel, Pranjal S Chorya, Karan Patel

**Affiliations:** 1 Department of Pathology, Zydus Medical College and Hospital, Dahod, IND

**Keywords:** embryonic remnants, meckel's diverticulum, omphalomesenteric duct (omd), umbilical granuloma, vitelline duct

## Abstract

The remnants of the omphalomesenteric duct (OMD) can lead to various congenital anomalies, including Meckel’s diverticulum, cysts, and fistulas, which may require surgical intervention. The OMD remnants usually occur in pediatric age groups. Clinical presentation of the patients varies widely. Some patients may remain asymptomatic throughout their lives. Some others may have complications necessitating surgical treatment. Symptoms include abdominal pain, gastrointestinal bleeding, or signs of infection. Imaging techniques such as ultrasound or CT scans are required to evaluate the presence of cysts, fibrous band tissue and its extension, or other anomalies. These umbilical lesions need to be surgically removed after a thorough examination. The clinical and pathological characteristics of four cases of umbilical lesions, including ectopic gastrointestinal tissue, are shown.

## Introduction

The embryonic site that offers communication between the yolk sac and the midgut during fetal development is known as the omphalomesenteric duct (OMD). This is also known as the vitelline or vitellointestinal duct. In the physiological development of the embryo, OMD undergoes self-regression between the fifth and ninth week of gestation [1]. Lack of this self-regression leads to a range of anomalies such as Meckel's diverticulum, patent vitelline duct, fibrous band, sinus tract, umbilical polyp and cyst, enteric fistula with ileal intussusception prolapsing over the umbilicus, or hemorrhagic umbilical mass [2]. Discharge from the umbilicus in neonates, infants, and early childhood is usually due to a remnant of the vitelline duct or a patent urachus. There have been case reports of rarer anomalies such as an infected vestigial umbilical artery, persistence of the vitelline artery, and a fistula between the vermiform appendix and a patent vitelline duct [3]. These various types of rare OMD remnants often present as a polyp. After the cord is separated, it is seen as a glistening, cherry-red nodule. Unlike the umbilical granuloma, the umbilical polyp is a remnant of the vitelline duct and consists of small bowel mucosa. OMD remnants are occasionally presented with some complications like abdominal pain, rectal bleeding, intestinal obstruction, umbilical drainage, and umbilical hernia. The appearances of these symptoms are age-dependent, and these lesions are usually treated surgically. Most of these symptoms appear before the age of four years [4]. Umbilical granuloma and umbilical polyp are the most common causes of umbilical discharge in infants. These umbilical lesions may be benign or inflammatory, or may be anomalies of the umbilical cord, such as umbilical polyps containing heterotopic tissue (e.g., gastric or pancreatic tissue) or Meckel diverticulum. The distinction between the usually superficial umbilical granuloma and deep-seated OMD remnants is crucial for appropriate treatment.

## Case presentation

Four cases of remnants of OMD presenting as umbilical lesions are discussed. Case 1 involves a four-year-old male child. He presented with an umbilical swelling 1.0 cm × 1.0 cm. Case 2 was a four-year-old female child. She presented with an umbilical swelling 1.1 cm × 1.0 cm. Case 3 was a three-year-old male child presenting with an umbilical swelling 1.2 cm × 1.0 cm. Case 4 was a 10-year-old male presenting with an umbilical swelling 1.5 cm × 0.5 cm.

Further appropriate investigations and treatment with surgical excision were carried out. The details of signs, symptoms, diagnosis, and treatment of the disease are summarized in Table 1. The sonographic images are not available for inclusion in this report.

**Table 1:** Summary of clinical features, treatment, and laboratory evaluation of the case series IHC: immunohistochemistry

Case details	Case 1	Case 2	Case 3	Case 4
Age/sex symptoms	Four-year male; swelling of the umbilicus since birth	Four-year female; swelling of the umbilicus, watery discharge	Three-year male; swelling of the umbilicus since birth	Ten-year male; swelling of the umbilicus since birth
Clinical examination	Umbilical swelling, 1.0 cm × 1.0 cm; associated with a discharge	1.1 cm × 1.0 cm growth umbilicus, watery discharge, no bleeding	1.2 cm × 1.0 cm growth umbilicus, serous discharge	1.5 cm × 0.5 cm growth umbilicus, minimal bloody discharge
Clinical diagnosis	Umbilical granuloma	Umbilical granuloma	Umbilical granuloma	Umbilical granuloma
Sonography examination	Hypoechoic collection, 12 mm × 6 mm; umbilical granuloma	A 7 mm × 6 mm hypoechoic superficial umbilical nodule; umbilical granuloma	8.8 mm defect midline anterior abdominal wall, umbilical region; possibility of umbilical hernia	Ill-defined hypoechoic collection 14 mm × 11 mm umbilical region; possibly infective
Surgery	Excision of umbilical tissue	Excision of umbilical tissue	Umbilical herniotomy; resection of granulation tissue	Excision of umbilical granulation tissue
Gross examination	Multiple tissue bits; 1.5 cm × 1.0 cm	Partially skin-covered grayish-white tissue bits; 1.0 cm × 0.8 cm	Nodular grayish white tissue bits; 0.8 cm × 0.6 cm	Grayish white tissue, partially skin-covered specimen; 0.5 cm × 0.3 cm
Histopathological examination	Squamous epithelium-lined skin, along with columnar epithelium-lined granulation tissue and fibrous stroma (Figure 1)	Polypoidal growth, intestinal epithelium, few columnar glands, connective tissue stroma; surrounding skin lining (Figure 2)	Polypoidal tissue, intestinal epithelium, columnar glands surrounding stratified squamous epithelium-lined skin (Figure 3)	Nodular structure lined by skin and intestinal epithelial glands, fibrocollagenous stroma, mixed inflammatory infiltrates (Figure 4)
IHC findings (glands)	CK7 negative, CK20 positive	CK7 positive, CK20 positive	CK7 positive, CK20 positive	CK7 negative, CK20 positive
Diagnosis	Remnants of the omphalomesenteric duct - umbilicus	Remnants of the omphalomesenteric duct - umbilicus	Remnants of the omphalomesenteric duct - umbilicus	Remnants of the omphalomesenteric duct – umbilicus

**Figure 1:**
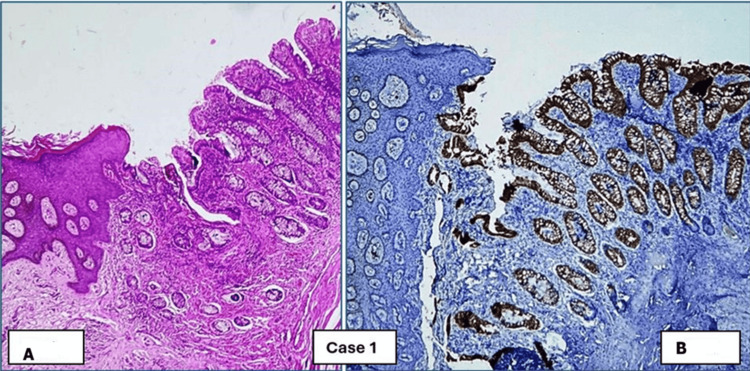
Remnants of omphalomesenteric duct in Case 1: umbilicus, CK7-negative (not shown), and CK20-positive. (A) H&E (4× magnification). (B) CK20 (4× magnification) H&E: hematoxylin and eosin

**Figure 2:**
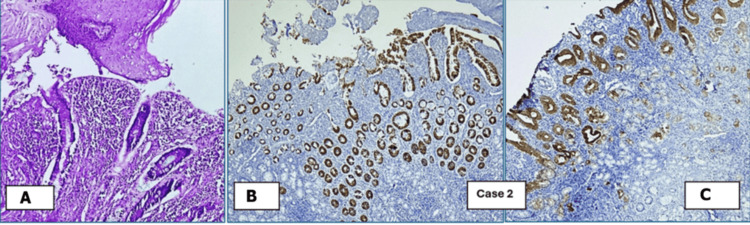
Remnants of omphalomesenteric duct in Case 2: umbilicus, CK7, and CK20 positive. (A) H&E (10× magnification). (B) CK7 (4× magnification). (C) CK20 (4× magnification) H&E: hematoxylin and eosin

**Figure 3:**
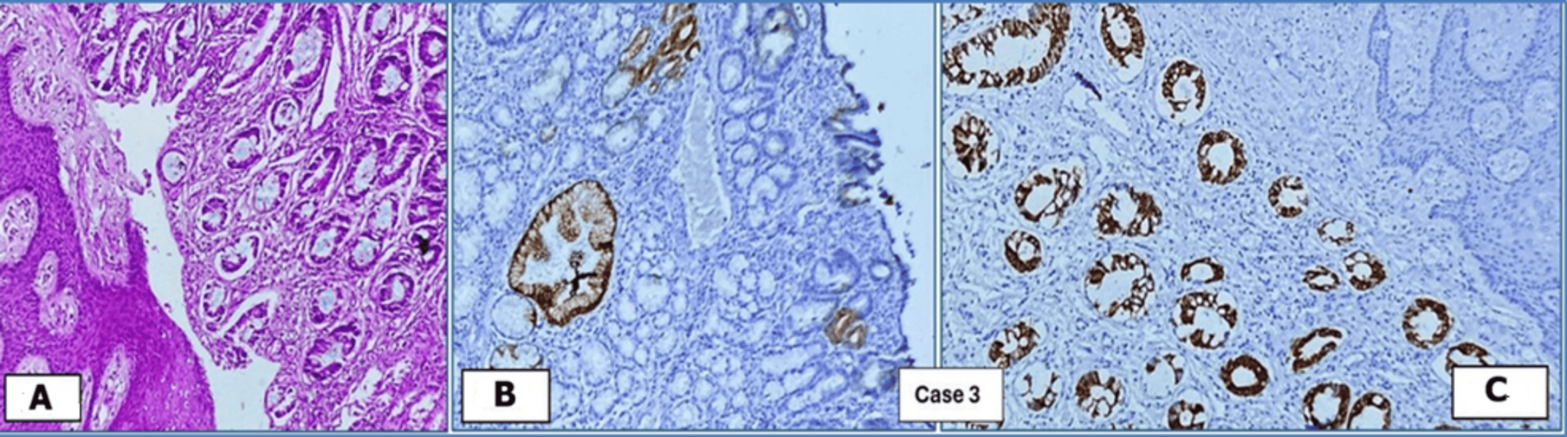
Remnants of omphalomesenteric duct in Case 3: umbilicus, CK7, CK20 positive. (A) H&E (10× magnification). (B) CK7 (10× magnification). (C) CK20 (10× magnification) H&E: hematoxylin and eosin

**Figure 4:**
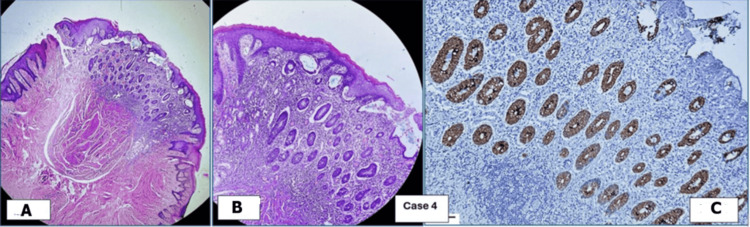
Remnants of omphalomesenteric duct in Case 4: umbilicus, CK7 negative (not shown), CK20 positive. (A) H&E (4× magnification). (B) H&E (10× magnification). (C) CK20 (10× magnification) H&E: hematoxylin and eosin

Histopathological and immunohistochemical (IHC) examinations of the various tissues received in the laboratory were performed. The microscopic images are presented below. Figures 1, 4 show remnants of OMD, which are CK20 positive. CK7 was negative in these cases, but it is not shown in the images. Figures 2, 3 show remnants of OMD, which are both CK7- and CK20-positive.

## Discussion

Remnants of OMD

The remnants of OMD are rare and occur postnatally in about 2% of infants. There is currently no known genetic basis for this condition [5]. The umbilical stump typically sheds between 5 and 15 days of life, following which normal skin covers the exposed portion of the surface. The raw area might occasionally bleed easily, along with some watery secretions, and develop a structure like a polyp that can be mistaken for an umbilical granuloma [1]. It is crucial to remember that the treatment of umbilical polyps and umbilical granulomas is different. Umbilical granulomas are typically treated with silver nitrate cauterization, ligation, or salt to dry them out, usually requiring no surgery. Conversely, umbilical polyps (rare, firm, red, congenital remnants) require surgical excision because they do not respond to conservative measures. Imaging modalities may be required to distinguish between clinical differentials. Umbilical endometriosis is an important differential diagnosis in females, and it may present as a mass or with discharge. Umbilical polyps typically also have ectopic small intestinal mucosa, although other ectopic tissues, e.g., gastric mucosa and pancreatic tissue, are quite rare [6,7].

Because OMD clinically resembles umbilical granuloma, its persistence may lead to incorrect clinical diagnoses. Other potential causes of umbilical lesions include urachus and omphalocele, Meckel hernia, umbilical hernia, umbilical remnants of the urachus, benign soft-tissue mass (e.g., epidermoid cyst, hemangiomas), and other benign soft-tissue tumors. It may also infrequently have heterotopic gastric and pancreatic tissue [8].

The theoretical basis for the presence of ectopic tissues in umbilical polyps has been postulated by the following reasoning. The three influential pathogeneses include misplacement theory, in which embry­onic tissue is located in an inappropriate place and develops into mature pancreatic tissue; metaplasia theory, stating that endodermal tissues migrate to the submucosa during embryo­genesis and transform into pancreatic tissue; and the totipotent cell theory, in which totipotent endodermal cells lining the gut or OMD differentiate into pancreatic tis­sue. In the metaplastic theory, the mid-gut rotates 90° counterclockwise along the umbilical cord, around the superior mesenteric artery, and extends to become the ileum and jejunum throughout embryonic development. At this stage of embryogenesis (10th week), the lumen of the OMD closes and the midgut is reinvested into the abdominal cavity. Intestinal and gastric mucosal cells may be implanted at this time at ectopic locations of pancreatic cells [8,9].

Intestinal tissue and heterotopic tissue can be found in umbilical polyps. Ectopic gastric or pancreatic cells in the remnants of the OMD may extend through the stalk and into the abdominal cavity when umbilical polyps are present. The tissues are vulnerable to the same pathological changes as their original organs, including neoplastic transformation, which must be ruled out. The gastric epithelium may produce gastric acid, leading to focal ulcers, secondary infections, and omphalitis. Ectopic pancreatic tissue, which has endocrine and exocrine functions, can cause extensive damage when released from the gastrointestinal tract. Therefore, various diagnostic modalities may be required to ascertain the completeness of the surgical resection.

IHC analysis was also done in these cases. Operating characteristics of the traditional combination of CK7 and CK20 markers as dichotomous (+/−) variables guide us to establish with reasonable certainty the origin of the heterotopic/ectopic tissue. CK7-negative, CK20-positive tissues normally indicate tissue of the lower gastrointestinal tract or colorectal origin. CK7-positive tissues with varying expression of CK20 status would favor an upper gastrointestinal tract origin.

In the present study, cases 1, 2, and 4 were clinically diagnosed as umbilical granulomas. However, histopathological examination revealed remnants of the OMD. In case 3, an umbilical defect was observed in addition to the umbilical growth and an umbilical hernia. These umbilical abnormalities appeared in early childhood, complicating the differential diagnosis of congenital OMD anomalies.

Clinically, the lesions presented as discharging umbilical granulomas; histopathology confirmed intestinal tissue, highlighting that although remnants of OMD are rare, they must be considered in the differential diagnosis of umbilical lesions. Complete surgical excision is the treatment of choice for appropriate clinical management.

## Conclusions

Although rare, remnants of the OMD presenting as umbilical polyps with ectopic gastrointestinal tissue should be considered in the differential diagnosis of persistent umbilical discharge and granulomas. Complete excision of such lesions is essential, followed by histological evaluation to confirm the absence of intra-abdominal extension and to identify ectopic tissues. These tissues are prone to the same pathological changes as their native organs, including ulceration, secondary infection, omphalitis, and potential neoplastic transformation, all of which must be excluded.
